# Annual Meeting at St. James's Palace

**Published:** 1916-12-23

**Authors:** 


### THE LEAGUE OF MERCY.

### Annual Meeting at St. James's Palace.

The annual meeting of Presidents of the League of Mercy was held at St. James's Palace on Tuesday, December 19, under the presidency of the Right Hon. the Lord Farquhar, G.C.V.O., when H.R.H. Princess Alexander of Teck was present, and handed the Order of Mercy to a large number of ladies and gentlemen in recognition of their work for the organisation.

### Letter from Their Majesties.

Buckingham Palace,
December 18, 1916.

My Dear Farquhar,—I am commanded by the King and Queen to assure all the officers and members that their Majesties continue to take a lively interest in the progress and welfare of the League of Mercy.

The King and Queen are pleased to learn that, besides again handing over £14,000 to King Edward's Hospital Fund, the League has distributed more than £3,000 to hospitals outside London, but within the area of collection.

This prosperity under war conditions appears to justify the Grand President and Council in having decided, in August 1914, to utilise the organisation of the League for assisting voluntary hospitals admitting wounded soldiers.

To have benefited the voluntary hospitals in and around London by contributions of more than £266,000 in eighteen years must be a source of sincere and genuine satisfaction to those who have laboured so self-sacrificingly to bring about that result, and their Majesties heartily congratulate the Presidents, Lady Presidents, and all those associated with them on their very successful efforts.

Yours very truly,
(Signed) Stamfordham.

### The Chairman's Speech.

Alluding to one paragraph in His Majesty's letter, Lord Farquhar thought that if the Grand President and Council had not decided, in August 1914, to utilise the organisation of the League of Mercy for assisting voluntary hospitals, the League would not have been in the present financial position, one which he thought the Treasurer and Hon. Secretaries would agree was very fortunate. In taking the chair by command, he keenly felt what was the real occasion for it—namely, the absence of the Grand President, Prince Alexander of Teck, caused by his fulfilling his duties at the Front, a fact which he, the speaker, deplored as much as anybody. They congratulated themselves very much on the presence of H.R.H. Princess Alexander of Teck, who had unfailingly done all she possibly could for the League for many years, and who would present the Order of Mercy to those to whom it had been awarded. And there were very few Lady Presidents who had been so successful as H.R.H. Princess Victoria and Princess Marie Louise of Schleswig-Holstein, Princess Victoria being Lady President of four counties—Berkshire, Buckinghamshire, Hampshire, and Oxfordshire—while Princess Marie Louise was Lady President of North Kensington district.

The year under review had been as remarkable as last year for the way in which British philanthropists had opened their pockets for the cause of the wounded soldiers; and the League had had to adopt new systems in order to keep up its large subscription to the hospitals. One of these schemes was the restaurant scheme, which was initiated by Captain and Mrs. Maclean. The second was a collection in the markets and exchanges of the City of London, which method originated with Mr. Bruce Shipway, the City Secretary. The third was the house-to-house envelope collection, which had been very successful, and was still pursuing its course. The fourth was one in which he was specially interested, because it had brought a very prosperous result in West Marylebone—namely, the film—and towards this good result Miss Andrews and her father had contributed largely. Beyond those various schemes the work had been carried on with undiminished success, and the results, considering the very many appeals now being made to the charitable, gave cause for much congratulation. He considered it would be a good thing if the Lady Presidents and Vice-Presidents were to arrange to have more meetings during the current year, and he was sure the indefatigable Secretary would do all he could to bring a speaker, and encourage such meetings in every way. The hospitals were asking for more and more funds, and he hoped they would get every shilling they needed. Up to the end of last month more than 120,000 sailors and soldiers had passed through those hospitals which were subsidised, directly or indirectly, through the funds of the League of Mercy. The Treasurer would be able to say how very acceptable these subsidies had been in these anxious times. Sir William Collins had told him that the State contribution to the wounded was 4s. per day, but that was not much more than half what they cost the hospitals. In one hospital which he knew, although the increase in the number of in-patients had been 10 per cent., the cost of the full diets had risen 30 per cent., owing partly to the robust appetites of the sailor and soldier patients, and partly to the enhanced prices of foods. He referred with much regret to the loss of one of the League's oldest workers, Lady Faudel-Phillips, the Lady President of the Hertford district. But he was glad to say her daughter had consented to carry on the work. He concluded by tendering to all the workers for the League the Council's gratitude for their continued and very successful efforts during a very trying year.

Sir Frederick Green (Hon. Treasurer) said it afforded him great pleasure to submit the annual statement of the results of the League's work, because those results were satisfactory, and reflected the greatest credit upon their workers in every grade. The total receipts until that morning reached £19,950, as against £19,582 this time last year. Donations were received last year after the meeting, and the total was £19,896. This year that total had already been passed, and further donations were sure to be received, as some of the envelopes had not yet come in. They felt justified in asking the King's Fund to allow the League to increase their donation to £15,000, which would raise the total contributions of the League to the King's Fund to £245,000. Besides the £15,000, they had given £3,577 to extra-Metropolitan hospitals, and in that sum was included £216 which had been returned to their branch in India, so that the Committee there might allocate it to the hospitals which they thought most deserving. There could be no doubt that the letter which was issued by the command of the Grand President at the beginning of the war had a very stimulating effect upon the branches. The two years which had elapsed since it was issued had yielded a thousand pounds more than the League received before it was circulated: previously there had been some falling off in the response. He urged his hearers to help to bring back the takings of the League to the splendid figure reached in 1908—namely, £22,000. There seemed no good reason why that should not be done even now.

### How this Year's Money was Raised.

The Treasurer proceeded to refer in more detail to the various new schemes which had been operative, as mentioned by the Chairman. The market collection had been greatly assisted by the then Lord Mayor, Sir Chas. Wakefield, who also defrayed the expenses. The Leadenhall Market, Lloyd's, and the Metal Exchange collection totalled £85; for this the League had to thank Mr. Hornby Steer. The Billingsgate Market £78, including £47 for a barrel of herrings put up for auction several times. The men entered most heartily into the spirit of the occasion. Lady Alexander collected at the Baltic Exchange £63. At the Central Meat Market the collection was over £60. The whole market collection totalled £417. The restaurant collection, inaugurated by Captain and Mrs. Maclean, reached £625, and a feature of that collection was the extraordinary amount of copper money. Lady Alexander collected over £90 in coppers. The League of Mercy film was shown in more than twenty districts throughout the country, and with the aid of numerous friends, especially Mrs. Fowler Penny, over £1,000 had been received from this source. The Council had been called upon to decide whether it should receive portions of the takings from Sunday cinema-shows. Ultimately the Council decided that as they were doing so well with the present shows it was better to leave well alone. They were, however, none the less grateful to those who had offered part of these Sunday takings. £750 was added by the envelope collection, and as more was still to come from this source it was still hoped that the total for this year would reach £20,000. Reading district had collected £120, Paddington £110. In one district a little girl aged twelve, a cripple, collected a large sum of money, and in another a blind lady did exceedingly well. The pastoral play given at Reading was a great success; it was honoured by the patronage of H.R.H. Princess Victoria and the effort yielded £270. The following districts each yielded over £500 and were mentioned in the order of merit: West Marylebone, £1,125; South Paddington, £1,103; South Kensington, £1,081; Berkshire, £960. Here the envelope collection yielded £128. Epsom and Esher, £833; St. George's, Hanover Square, £614, included in which was a cheque for 100 guineas from Colonel Hamilton; Hampstead, £580. West Marylebone was £400 better than last year, South Paddington £204, Berkshire £326, Battersea £204, East Marylebone £54, Strand £172, South St. Pancras £169, Watford £66, East Grinstead £61, Uxbridge £82.

